# Study on the Material Properties of Microconcrete by Dynamic Model Test

**DOI:** 10.3390/ma15103432

**Published:** 2022-05-10

**Authors:** Chunyu Zhang, Jinpeng Zhang, Qichao Ren, Jianguo Xu, Bo Wang

**Affiliations:** 1 School of Water Conservancy Engineering, Zhengzhou University, Zhengzhou 450001, China; zcy18334930965@gs.zzu.edu.cn (C.Z.); rqcsilence@163.com (Q.R.); wangbo@zzu.edu.cn (B.W.); 2 Henan Puze Expressway Company Limited, Puyang 457000, China; 202022222014451@gs.zzu.edu.cn

**Keywords:** aqueduct structure, scaled model test, orthogonal experiment, microconcrete, mechanical properties

## Abstract

As an important water conveying structure, the seismic safety of the hydraulic aqueduct has attracted considerable interest. Different from the general bridge structure, the seismic analysis of the aqueduct structure needs to consider its fluid–structure interaction. The existing numerical simulation methods cannot truly reflect the fluid–solid coupling mechanism. Therefore, scholars began to use shaking table tests to study the fluid–structure interaction mechanism. However, the research is immature, and it is mostly focused on the seismic response analysis, and there are few studies on the model test similarity ratio and model material properties. Based on this, in this paper, according to the requirements of the test similarity ratio, the orthogonal experiment was used to explore the influence of barite sand content, water–cement ratio, fine sand ratio, and lime ratio on the mechanical properties of microconcrete. The performance indicators of microconcrete under different mix ratios vary widely, with a minimum variation of 19% and a maximum of 102%. Barite sand has the most significant control effect on the density, and the water–cement ratio has the most significant control effect on the compressive strength and elastic modulus. The density variation range is 2.37–2.81 g/cm^3^, the cube compressive strength variation range is 18.37–36.94 MPa, and the elastic modulus variation range is 2.11 × 10^4^–3.28 × 10^4^ MPa. This study will provide certain evidence for the similarity ratio design and material selection of the scaled model test of the fluid–solid coupling structure.

## 1. Introduction

As an important water conveyance structure, the hydraulic aqueduct is widely used in long-distance water transfer projects. A large number of reinforced concrete aqueduct structures were used in the South-to-North Water Diversion Project, some of which are distributed in areas with high seismic intensity. Strong earthquakes can easily cause the interruption of the aqueduct water transmission line, and it is very likely that local secondary disasters will occur due to the short-term concentrated dumping of water bodies. The aqueduct is also more difficult to repair after an earthquake than other traffic bridge structures. Therefore, the seismic safety of the large-scale hydraulic aqueduct structure has attracted great attention. Under the action of an earthquake, in addition to static forces, the bottom and walls of the aqueduct are also subject to hydrodynamic pressure. This fluid–solid dynamic coupling problem is very complicated, and it is very unfavorable for the seismic resistance of the aqueduct structure. Therefore, how to correctly simulate the dynamic interaction between the water and the aqueduct is the key to the seismic design of the aqueduct. At present, in the seismic analysis of aqueducts, the Housner model is generally used to simulate the water in the aqueduct. This model ignores the deformation of the aqueduct structure, assumes that the tank is rigid, and does not consider the elastic vibration of the tank wall, so the applicability is limited [1,2,3,4,5]. At the same time, many scholars use mathematical models, such as the boundary element method and finite element method, to study the dynamic interaction between water and aqueduct [6,7,8], which can reflect the influence of fluid sloshing on the structure. However, this presents higher requirements in respect of the motion algorithm of the grid. Due to the complexity of the fluid–solid coupling mechanism, a digital–analog method that can truly and reasonably reflect the fluid–solid coupling mechanism has not yet been developed. Therefore, scholars have begun to conduct shaking table model scaled tests on aqueducts, hoping to provide a basis for rationally simulating the dynamic effects of water bodies in the seismic calculation of aqueducts.

The earthquake simulation shaking table test is currently an important and effective method for studying the seismic response, failure mechanism, seismic reliability, and damping effect of engineering structures. At present, only a few scholars have conducted shaking table test research on aqueduct structures. In the early research on the shaking table of the aqueduct, the study was biased towards the seismic response of the aqueduct structure. The dynamic characteristics and seismic response characteristics of aqueduct structures of different types of aqueducts under the action of horizontal or vertical earthquakes are discussed [9,10,11]. In addition, some scholars have also explored the application effects of seismic isolation technology in aqueduct structures through shaking table tests [12,13,14]. For the structural scale model test, the similarity between the model material and the original structure material has a significant impact on the final result of the model test. Therefore, choosing the appropriate test material is a key issue for the success of the shaking table test. At present, for the shaking table test of the aqueduct structure, in order to meet the material similarity ratio requirements, rubber or plexiglass are mostly used [15,16,17,18,19,20,21]. Due to the large plasticity and Poisson ratios of the two materials, they are not suitable for destructive dynamic tests. Ordinary concrete cannot be poured because the particle size is too large. Therefore, the use of micro-concrete with similar mechanical properties to make a model can effectively solve the above problems. Existing studies focus on the strength properties of microconcrete [22,23,24,25,26,27], ignoring studies on density properties. In the model test of the aqueduct structure, the fluid–structure interaction should be considered, so it is necessary to adjust the density of micro-particle concrete to satisfy the similarity ratio relationship of the aqueduct structure model. Based on this, this paper innovatively proposes to use barite sand and quartz sand with a large difference in density to adjust the density of microconcrete. Moreover, the orthogonal test design method is proposed to study the influence and sensitivity of different factors on the performance of microconcrete materials, in order to provide relevant basis for the application of micro-concrete in the shaking table test of the aqueduct structure model.

## 2. Model Material Similarity Theory

The hydraulic destructive dynamic model test is usually a scale model test, which requires the model material to be weakened accordingly to achieve the similarity between the prototype and the model. That is, the corresponding physical quantities of the prototype and the model have a certain proportional relationship and follow the same mechanics law.

According to the equation analysis method, the similar conditions of the structure can be determined. From the theory of elasticity, the similar conditions of materials in the elastic range of static structure can be deduced. Use the superscript ′ to indicate the corresponding physical quantity of the model, which has similarity coefficients:

$$\frac{x}{x^{\prime }}=C_{l};\;\frac{u}{u^{\prime }}=\frac{u_{0}}{{u^{\prime }}_{0}}=C_{w};\;\frac{\varepsilon }{\varepsilon^{\prime }}=\frac{\gamma }{\gamma^{\prime }}=C_{\varepsilon };\;\frac{p}{p^{\prime }}=C_{P};\;\frac{\sigma }{\sigma^{\prime }}=\frac{\tau }{\tau^{\prime }}=C_{\sigma };\;\;\frac{E}{E^{\prime }}=C_{E};\;\frac{\rho_{x}}{\rho_{x}^{\prime }}=C_{\rho }$$

Substituting the above quantities into the basic equation expressions of each prototype, they should be the same as the corresponding equations of the model. For example, the x-direction balance equation of the prototype structure:

(1) $$\frac{\partial \sigma_{x}}{\partial x}+\frac{\partial \tau_{xy}}{\partial y}+\frac{\partial \tau_{xz}}{\partial z}+\rho_{x}=0$$

Substituting the similarity coefficient can get:

(2) $$\frac{C_{\sigma }}{C_{l}}\left(\frac{\partial \sigma_{x}^{\prime }}{\partial x^{\prime }}+\frac{\partial \tau_{xy}^{\prime }}{\partial y^{\prime }}+\frac{\partial \tau_{xz}^{\prime }}{\partial z^{\prime }}\right)+C_{\rho }\rho_{x}^{\prime }=0$$

This should be the same as the model structure balance equation, so we can get:

(3) $$\frac{C_{\sigma }}{C_{l}C_{\rho }}=1$$

In the same way, by the geometric equation:

(4) $$\begin{array}{l} C_{\varepsilon }\varepsilon_{x}^{\prime }=\frac{C_{w}}{C_{l}}\frac{\partial u^{\prime }}{\partial x^{\prime }}\\ C_{\varepsilon }\gamma_{xy}^{\prime }=\frac{C_{w}}{C_{l}}\left(\frac{\partial u^{\prime }}{\partial y^{\prime }}+\frac{\partial \nu^{\prime }}{\partial x^{\prime }}\right) \end{array}$$

This should be the same as the model structure geometric equation, so we can get:

(5) $$\frac{C_{\omega }}{C_{l}C_{\varepsilon }}=1$$

From the physical equation:

(6) $$\begin{array}{l} C_{\varepsilon }\varepsilon_{x}^{\prime }=\frac{C_{\sigma }}{C_{E}}\frac{1}{E}\left[\sigma_{x}^{\prime }-C_{\mu }\mu^{\prime }\left(\sigma_{y}^{\prime }+\sigma_{z}^{\prime }\right)\right]\\ C_{\varepsilon }\gamma_{xy}^{\prime }=\frac{C_{\sigma }}{C_{E}}\frac{2\left(1+C_{\mu }\mu^{\prime }\right)}{E^{\prime }}\tau_{xy}^{\prime } \end{array}$$

This should be the same as the model structure physical equation, so we can get:

(7) $$\frac{C_{\sigma }}{C_{E}C_{\varepsilon }}=1,C_{\mu }=1$$

From the boundary conditions:

(8) $$\begin{array}{l} C_{p}p_{x}^{\prime }=C_{\sigma }\left(\sigma_{x}^{\prime }l+\tau_{xy}^{\prime }m+\tau_{xz}^{\prime }n\right)\\ C_{\omega }u^{\prime }{|}_{s}=C_{\omega }u_{0}^{\prime } \end{array}$$

This should be the same as the model structure boundary conditions, so we can get:

(9) $$\frac{C_{\sigma }}{C_{p}}=1$$

In fact, under the condition of small elastic deformation, the stress has nothing to do with the elastic modulus. It only needs to satisfy: similar geometry, similar load, similar boundary conditions, and the same Poisson’s ratio. It does not need to satisfy Hooke’s similarity at the same time, that is, generalized similarity. In this way, among the eight similarity coefficients, the Poisson’s ratio should be the same, and there are still seven similarity coefficients. Subject to the constraints of the three equations, three similarity coefficients can be freely selected as the basic similarity ratio for the model test. Compared with the static structure, the material similarity requirement in the dynamic test has an additional damping coefficient ζ, and there has been no unified equation for the theory of damping coefficient, and the dimension is not unique, so it is generally not considered. In addition, the dynamic test also needs to meet the similar test conditions, that is, the acceleration and time are similar. Different from the bridge structure, the shaking table model test of the aqueduct structure needs to consider the effect of the water body due to the fluid–solid coupling effect, so $C_{\rho }=1$ is required. Secondly, the size and acceleration loading conditions of the vibrating table itself should also be considered.

## 3. Performance Test of Microconcrete

Microconcrete is a mixture of cement, water, and artificially prepared aggregates of less than 5 mm in an appropriate ratio. It has mechanical properties similar to ordinary concrete, and can reasonably simulate anisotropy and the bonding between the material and the steel bar. It is an ideal model test material. The appropriate particle size gradation and mixing ratio represent the key to the success of the structural model test. Existing research focuses on the similarity relationship between the strength of microconcrete and prototype concrete [17,18,19,20,21], ignoring the similarity of the density of the two. The aqueduct structure model test is different from other model tests because it is difficult to find a fluid instead of water to fully satisfy the density similarity ratio. Therefore, the density of the microconcrete needs to be adjusted to meet the similarity ratio relationship of the aqueduct structure model. In view of this research background, this paper uses different mass ratios of barite sand and quartz sand to prepare microconcrete to study the influence of different factors on the density, strength and elastic modulus of microconcrete. This provides a certain basis for the design of the model similarity ratio of the shaking table test of the fluid–solid coupling structure.

### 3.1. Raw Material Selection

In consideration of the density of microconcrete, barite sand and quartz sand with obvious difference in density index are selected as aggregate. The aggregate size should meet the requirements of similarity ratio, protective layer thickness, and steel bar spacing. Among them, the coarse aggregate particle size range is 2.5–5.0 mm, and the fine aggregate particle size range is 0–2.5 mm. The aggregate gradation should be continuous to control the discreteness and workability of concrete performance. However, the fine aggregate passing through the smallest sieve should be properly controlled to avoid excessively increasing the water–cement ratio. The particle gradation test results of the two materials are shown in Table 1. P.O.42.5 Ordinary Portland Cement is used as the cementing material, and the performance indicators of the cement material are shown in Table 2. Lime is used as the regulator.

### 3.2. Experimental Design

**Figure 1:**
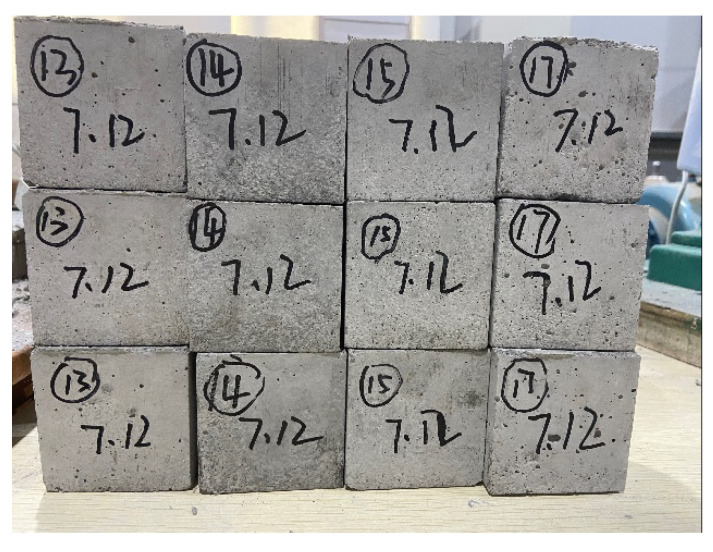
Specimen of microconcrete.

In this paper, the orthogonal test design method [28,29] is used to study the influence of four factors, namely water–cement ratio, barite sand content, fine sand ratio, and lime content on the mechanical properties of microconcrete. The content of barite sand (100% × $m_{barite\;sand}/m_{aggregate}$) is represented by A, and five levels are 25%, 40%, 50%, 63%, and 75%. The water–cement ratio is denoted by B. According to “General Concrete Mixture Design Regulation JGJ55-2011”, the water–cement ratio of concrete takes four levels of 0.4, 0.6, 0.8, and 1.0. The ratio of fine sand (100% × $m_{fine\;aggregate}/m_{aggregate}$) is represented by C, that is, the proportion of fine aggregate in barite sand and quartz sand in the total aggregate. When the coarse aggregate particle size is less than 10 mm, according to different water–cement ratios, four levels of fine sand ratio are 30%, 40%, 50%, and 60%. The lime content ($m_{lime}/m_{cement}$) is denoted by D, with four levels of 0, 0.2, 0.4, and 0.6. According to the orthogonality, select some representative points to carry out the test. The main tool of the orthogonal test is the orthogonal table $L_{n}\left(t^{c}\right)$. Use L as the code of the orthogonal table, n is the number of experiments, t is the level number, and c Is the number of columns, that is, the number of factors that can be arranged the most. The number of levels in each column may not be equal, which is called a hybrid orthogonal table. This experiment uses a hybrid orthogonal table L_20_ (5 × 4^3^) to determine the mix ratio combination of microconcrete, which means that 20 experiments are needed, and up to four factors can be observed. Among them, one factor is 5 levels, and the other factors are 4 levels. The specific test design is shown in Table 3. According to the mix ratio, a total of 20 groups of microconcrete need to be produced. Because the particle size range of the aggregate particle size of the microconcrete and mortar is close, according to “JGJ/T70-2009 Standard for Basic Performance Test Method of Building Mortar”, 3 pieces of 70.7 mm × 70.7 mm × 70.7 mm cube specimens and 6 pieces of 150 mm × 150 mm × 300 mm prism specimens were made for each group of mix ratio. Some test specimens are shown in Figure 1.

### 3.3. Test Method

**Figure 2:**
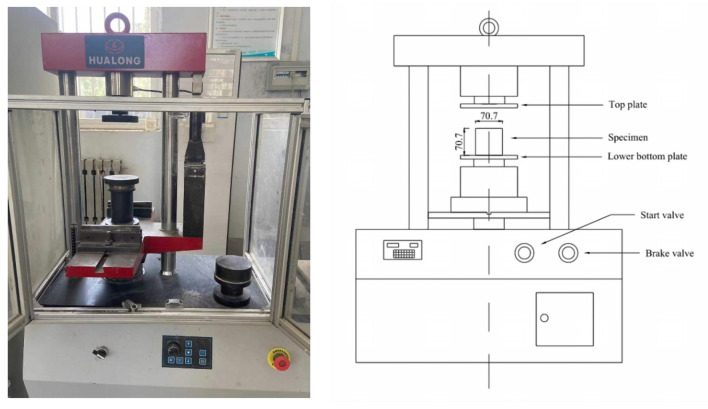
Compressive Strength Test.

The compressive strength test of the microconcrete cube was carried out on the WHY-300 computer-controlled pressure testing machine of Shanghai Hualong Company (Shanghai, China). The maximum load of the equipment is 300 KN, and it can be loaded in control modes, such as load and displacement. The test device and schematic diagram are shown in Figure 2. Place the specimen on the lower platen of the testing machine, align the center of the specimen with the center of the lower platen of the testing machine, start the testing machine, and apply the load continuously and evenly. When the specimen begins to deform sharply, stop adjusting the testing machine and record the failure load.

**Figure 3:**
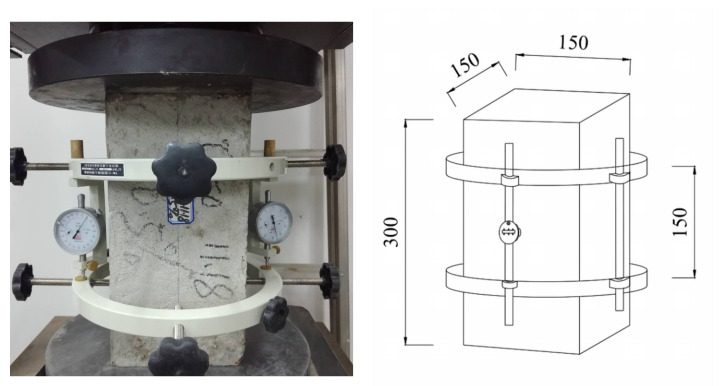
Elastic modulus test.

The elastic modulus is measured by an elastic modulus tester (Shengshihuike, Shanghai, China) and a compression testing machine (Hualong, Shanghai, China). The elastic modulus tester and the test process of the specimen are shown in Figure 3.

**Figure 4:**
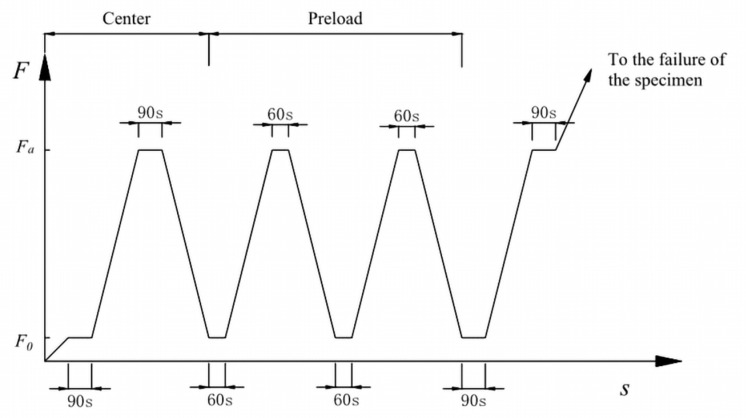
Schematic diagram of the loading process. Note: 90 s includes 60 s load application time and 30 s data recording time.

Take three specimens for the axial compressive strength test and calculate the axial compressive strength $f_{cp}$. The other three specimens were taken for the elastic modulus test, and the schematic diagram of the load application method is shown in Figure 4.

The elastic modulus should be calculated as follows:

(10) $$E_{c}=\frac{F_{a}-F_{0}}{A}\times \frac{L}{\Delta n}$$

(11) $$\Delta n=\varepsilon_{a}-\varepsilon_{0}$$

where $E_{c}$ denotes the elastic modulus of concrete under static pressure (MPa);

$F_{a}$ load when stress is 1/3 axial compressive strength (N);
$F_{0}$ initial load when stress is 0.5 Mpa (N);
A pressure bearing area (mm^2^);
L measuring gauge length (mm);
Δn the average value of the deformation on both sides of the specimen during the last loading from $F_{0}$ to $F_{a}$;
$\varepsilon_{a}$ the average value of the deformation on both sides of the specimen when the load is $F_{a}$;
$\varepsilon_{0}$ the average value of the deformation on both sides of the specimen when the load is $F_{0}$.

## 4. Analysis of Test Results

### 4.1. Full Curve of Compressive Load and Displacement

**Figure 5:**
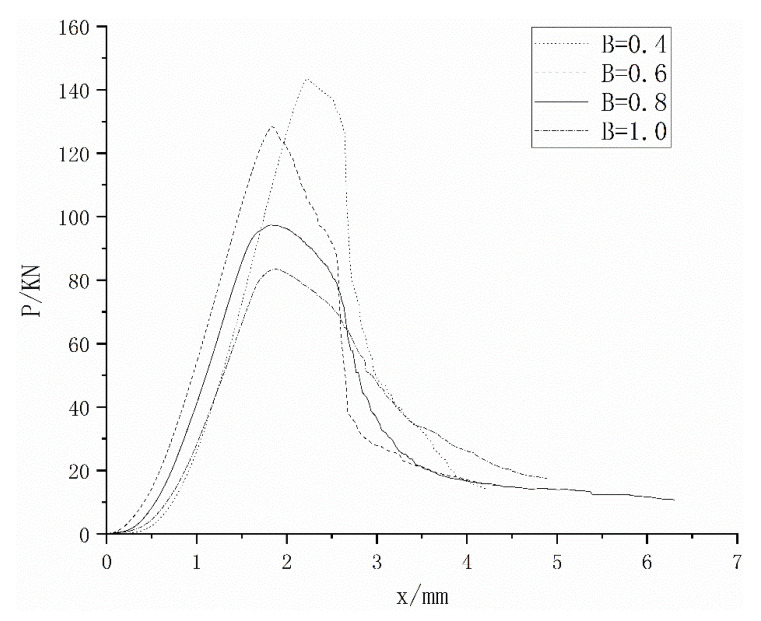
Curve of compressive load and displacement.

In the actual measurement of the compressive strength of the microconcrete cube, the full curve of compressive load and displacement is drawn, as shown in Figure 5.

Figure 5 shows the full curves of compressive load–displacement of microconcrete under different water–cement ratios. It can be seen from the figure that under different water–cement ratios, the change trend of the curve is basically the same, all rising first and then falling. When the displacement tends to infinity, the force on the specimen tends to zero. At the beginning of loading, the relationship between load and displacement is roughly linear. When the load exceeds 40% of the maximum load of the specimen, the slope of the curve decreases to zero. After the maximum bearing capacity is exceeded, the curve begins to drop, and the deformation of the specimen increases as the load decreases. There is a reverse bending point in the descending section, and the specimen still has a certain bearing capacity under greater deformation. It can also be seen from Figure 5 that as the water–cement ratio increases, the maximum bearing capacity of the specimen gradually decreases.

**Figure 6:**
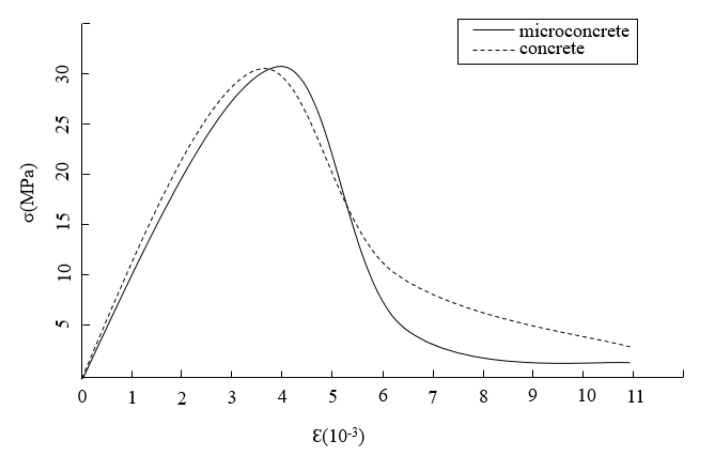
Stress-strain curve.

In the compressive strength test, the resistance strain gauge is pasted on the surface of the object to be measured, and the strain is converted into electrical parameters for testing. The stress is taken as the ratio of the load to the area of the specimen. The stress–strain curves of microconcrete and ordinary concrete with the same peak stress are shown in Figure 6. It can be seen from the figure that when the same peak stress is reached, the strain corresponding to microconcrete is greater than that of ordinary concrete. Therefore, the appearance of the fracture zone of microconcrete is later than that of ordinary concrete.

### 4.2. Analysis of Orthogonal Test Results

This test mainly studies the density, compressive strength, and elastic modulus of microconcrete. The test results are shown in Table 3. Orthogonal experimental design result analysis methods mainly include the direct comparison method and intuitive analysis method. The direct comparison method is a simple direct comparison of the test results, and some qualitative explanations are given to the test results, but the optimal results cannot be obtained. The intuitive analysis method is followed to carry out the range or variance analysis of the test results of different factors. The range analysis is followed to consider the influence of other factors on the result is balanced when considering the A factor and analyze the problem through the range value of each factor level. Using the range analysis of orthogonal test data, the influence of each factor on the test index can be sorted. According to Table 3 showing the results of this microconcrete test, the range analysis results of different influencing factors for microconcrete are shown in Table 4.

Analysis of variance, also known as “F test”, is used to study the relationship between one or more sub-type independent variables and a numerical dependent variable. The basic principle of analysis of variance relies on there being two types of data fluctuations. One is the difference caused by random errors in the experiment, called the intra-group difference SSE, which is the sum of squared errors between the sample data of each level or group and its group mean. The formula is shown in Equation (12). One is the difference caused by different test levels, called the difference between groups SSA, that is, the sum of squared errors between the mean of each group and the total mean. The calculation formula is shown in Equation (13).

(12) $$SSE=\sum_{i=1}^{k}\sum_{j=1}^{n_{i}}{\left(x_{ij}-{\bar{x}}_{i}\right)}^{2}$$

(13) $$SSA=\sum_{i=1}^{k}n_{i}{\left({\bar{x}}_{i}-\bar{x}\right)}^{2}$$

This paper conducts a single-factor analysis of variance. First, hypothesis H_0_: $\mu_{1}=\mu_{2}=\ldots =\mu_{k}$ is proposed, i.e., the independent variable has no significant effect on the dependent variable. Then the intra-group difference SSE and the between-group difference SSA are calculated, and they are finally divided by their corresponding degrees of freedom to get the mean square error. The specific calculation formula is shown in Equation (14). Then, F = MSA/MSE~F (k – 1, n – k).

(14) $$\begin{array}{l} MSA=SSA/k-1\\ MSE=SSE/n-k \end{array}$$

Among them, n is the number of all observations and k is the number of factor levels. At a given significance level α, calculate the F_α_ of the F (k – 1, n – k) distribution. If F > F_α_, the original hypothesis is rejected, indicating that the independent variable has a significant influence on the dependent variable. The results of the single-factor analysis of variance for the performance of microconcrete in this paper are shown in Table 5.

Taking the density index as an example, under the condition of confidence level α = 0.1, there are five levels of barite content A, and the critical value of factor significance F_0.1_(4,15) = 2.36, There are four levels of water–cement ratio B, fine sand ratio C and lime ratio D, and the critical value of factor significance is F_0.1_(3,16) = 2.46. In Table 5, F_A_ > 2.36, indicating that the amount of barite sand has a significant impact on the density of microconcrete; F_B_ > 2.46, F_C_ < 2.46, F_D_ < 2.46; indicating that the water–cement ratio B has a significant impact on the density of microconcrete; fine sand ratio C and lime ratio D have no obvious influence on the density of microconcrete. The analysis of other indicators is similar.

(1) Density

**Figure 7:**
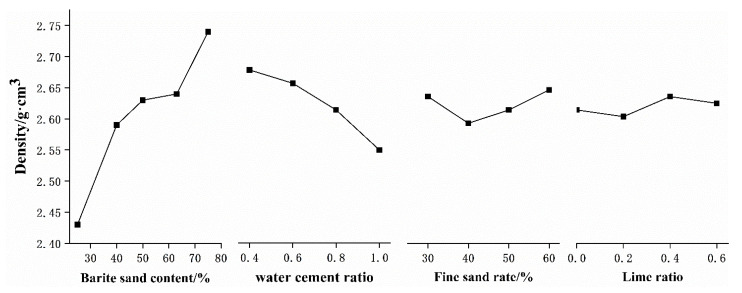
The relationship between different factors and the density of microconcrete.

From range analysis and variance analysis, it can be known that under the two analysis methods, the content of barite sand is the main factor controlling the density of microconcrete. The second is the water–cement ratio, while the fine sand ratio and the lime ratio are slightly different, but the impact on the density index is not obvious. In order to visually observe the influence trend of various factors on the density of microconcrete, the relationship between the different levels of each factor and the compressive strength of microconcrete is plotted in Figure 7.

Combining Figure 7 and Table 3, it can be seen that the content of barite sand has a significant effect on the density of microconcrete. The density increases with the increase of the content of barite sand. The content of barite sand increased from 25% to 75%, and the density increased by 12.76%. When the content of barite sand reaches 75%, the density of the microconcrete can reach up to 2.81 g/cm^3^. The range value of the water–cement ratio is close to the range value of the barite sand content, and the variance calculation result is significant, and the material density decreases with the increase of the water–cement ratio, because the increase in the water–cement ratio causes the pores of the microconcrete to become larger, thereby reducing the density. The fine sand ratio refers to the proportion of small grain size aggregates in barite sand and quartz sand, and the material density is mainly determined by the range of the particle framework established by the coarse aggregate. Therefore, the density of microconcrete is not sensitive to the fine sand ratio. Both the range and variance of the lime ratio are the smallest. The reason for this is that the density of slaked lime and cement are relatively close. Adjusting the proportion of slaked lime and cement cannot significantly change the density of microconcrete. In summary, how to select the amount of barite sand is the key to adjust the material density and eliminate the gravity effect of the model structure.

(2) Compressive strength

**Figure 8:**
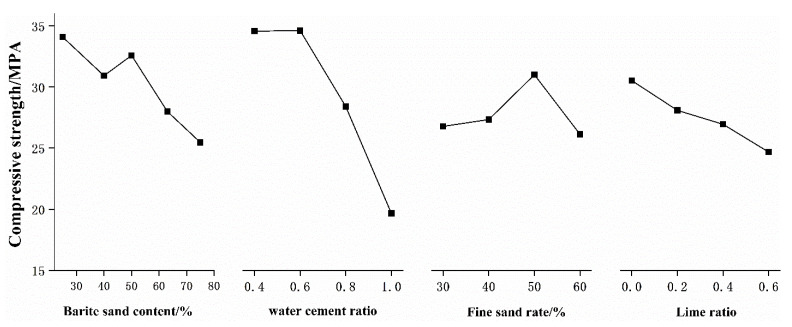
The relationship between various factors and the compressive strength of microconcrete.

From the range analysis in Table 4 and the variance analysis in Table 5, it can be seen that the order of the influence of various factors on the compressive strength of microconcrete is consistent, and they are: water–cement ratio > barite sand content > lime ratio > fine sand ratio. Under the two analysis methods, the water–cement ratio has the most obvious effect on the compressive strength, which is consistent with the existing research conclusions of concrete. The difference analysis results of barite sand content at different confidence levels are significant and insignificant respectively. Therefore, the content of barite sand can be used as the second controlling factor. In order to visually observe the influence trend of various factors on the compressive strength of microconcrete, the relationship between the different levels of each factor and the compressive strength of microconcrete is plotted in Figure 8.

It can be seen from Figure 8 that the lower the water–cement ratio, the higher the compressive strength. When the water–cement ratio is 0.4, the compressive strength is increased by 75.89% compared to when the water–cement ratio is 1.0. When the water–cement ratio is 0.4, the compressive strength is slightly lower than when the water–cement ratio is 0.5. This may be because the low water–cement ratio causes the concrete to not vibrate and compact, which easily causes the honeycomb pores in the concrete to increase, thereby reducing the strength. The compressive strength decreases with the increase of barite content. The compressive strength of 75% barite sand is 25.29% lower than that of 25% barite sand. The main reason for this is that the Mohs hardness of quartz sand is higher than that of barite sand, which indirectly leads to a decrease in the compressive strength of microconcrete. The ratio of fine sand has little effect on the compressive strength of microconcrete, and the maximum change range does not exceed 10%. The compressive strength decreases with the increase of the lime ratio. The incorporation of lime reduces the relative content of cement, resulting in insufficient binding force between the sand and the aggregate inside the test block, which reduces the compressive strength of the microconcrete. However, compared to other influencing factors, it is not particularly obvious.

(3) Elastic modulus

**Figure 9:**
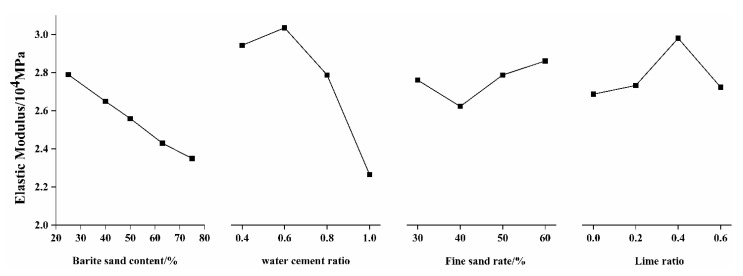
The relationship between various factors and the elastic modulus of microconcrete.

From the range analysis in Table 4 and the analysis of variance in Table 5, it can be seen that, under the two analysis methods, the water–cement ratio has the most obvious effect on the elastic modulus. The results of range analysis and variance analysis of fine sand ratio and lime ratio are slightly different, but the effect on elastic modulus is not significant. Therefore, the main factors controlling the elastic modulus are the water–cement ratio and the content of barite sand. In order to visually observe the influence trend of various factors on the elastic modulus of microconcrete, the relationship between the different levels of each factor and the elastic modulus of microconcrete is plotted in Figure 9.

It can be seen from Figure 9 that the change trend of the elastic modulus with the water–cement ratio is similar to that of the compressive strength, because the effect mechanism of the water–cement ratio on the elastic modulus is consistent with the strength. That is to say, the increase of the water–cement ratio makes the pores of the microconcrete increase, and the material bonding force decreases. When the fine sand ratio is at a low level, increasing the fine sand ratio can increase the compactness of the material, and the elastic modulus will increase accordingly. However, when the fine sand ratio is too large, the skeleton effect of the coarse aggregate is weakened, and the elastic modulus is reduced.

### 4.3. Destruction Morphology Analysis

**Figure 10:**
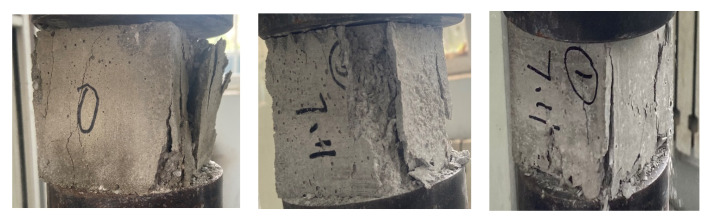

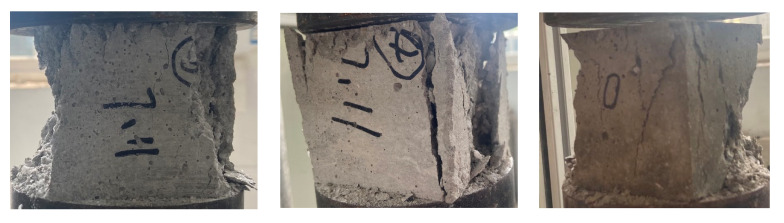
Different ratios of failure modes.

Figure 10 shows the compressive destruction morphology of the microconcrete cube specimens in different proportions. It can be seen from the destruction morphology of different specimens that the destruction morphology of microconcrete under different barite content is basically similar, and the water–cement ratio has the greatest impact on the destruction morphology. When the water–cement ratio is relatively small, it is difficult to vibrate the specimen evenly, and the surface of the specimen is easy to form honeycombs and pockmarked surfaces. The phenomenon of loose aggregate, broken particles, and slag falling occurs when it is destroyed. When the water–cement ratio increases, the surface of the test piece is smooth, the two ends of the test piece are seriously damaged, and the side edges appear peeling. Judging from the broken test block that was opened, the cracks were mainly present on the bonding surface of the aggregate and cement mortar and the inner surface of the cement mortar, and no aggregate cracks were found.

## 5. Conclusions

This article starts from the shaking table test of an aqueduct structure that needs to consider the effect of fluid–structure coupling. According to the requirements of the test similarity ratio, the orthogonal experiment was used to explore the influence of barite sand content, water–cement ratio, fine sand ratio, and lime ratio on the density, compressive strength, elastic modulus, stress–strain relationship, and failure morphology of microconcrete. In order to obtain the appropriate particle size gradation and mix ratio, this study will provide certain evidence for the model similarity ratio design of the shaking table test of the fluid–solid coupling structure of the aqueduct. The main conclusions reached are:

(1) It can be known from the experimental data that this orthogonal experimental level design can include the main influence interval, and the effect is relatively ideal. Using range analysis and variance analysis, it is concluded that the sensitivity of each factor to density is ranked as follows: barite sand content > water–cement ratio > fine sand ratio > lime ratio. The order of sensitivity of compressive strength and elastic modulus is: barite sand content > water–cement ratio > lime ratio > fine sand ratio. The stress–strain curve and failure form of microconcrete are the same as those of ordinary concrete, but the strain corresponding to the same peak stress is larger, and the width of the failure zone is smaller than that of ordinary concrete.
(2) The performance indicators of microconcrete under different mix ratios have a wide range of changes, with a minimum of 19% and a maximum of 102%. The density range is 2.37–2.81 g/cm^3^, the cubic compressive strength range is 18.37–36.94 MPa, and the elastic modulus range is 2.11 × 10^4^–3.28 × 10^4^ MPa. Moreover, the distribution range can be wider as the control factor level changes. A wide range of index distribution can better meet the model similarity ratio design and provide a reference for the preparation of different structural model tests of microconcrete.
(3) Barite sand has the most significant control effect on the density of microconcrete, and the water–cement ratio has the most significant effect on the compressive strength and elastic modulus of microconcrete. Therefore, to prepare microconcrete that meets the similar ratio conditions of the model shaking table test, it is first necessary to adjust the water–cement ratio to make the strength and elastic modulus of the microconcrete within the corresponding range, and then adjust the amount of barite sand to make the material density meet the density similarity condition of the fluid–solid coupling structure. Finally, the material properties are fine-tuned through the fine sand ratio, particle size gradation, and lime ratio to achieve the optimal mix ratio that meets the similarity ratio of the model.

## Figures and Tables

**Table 1:** Test results of aggregate gradation.

Aggregate	Mesh Size/mm					
	5.000	2.500	1.250	0.625	0.313	0.156
Barite sand	13.64%	52.18%	60.26%	74.69%	86.33%	96.43%
quartz sand	6.52%	43.72%	54.38%	69.27%	80.59%	95.87%

**Table 2:** Cement performance indicators.

Index	Standard Value	Test Value
Specific surface area/m^2^·kg^−1^	≥300	374
Initial setting time/min	≥45	171
Final setting time/min	≤600	217
Stability/mm	≤4	1.5
28-day compressive strength/MPa	≥42.5	45
28-day flexural strength/MPa	≥6.5	8.5
Standard consistency water consumption/%	27–31	27.4

**Table 3:** Proportioning scheme for orthogonal test of microconcrete.

Test Number	Influencing Factors				Mix Ratio	Density	Compressive Strength	Elastic Modulus
	A	B	C	D				
T-1	1	1	4	1	1:1:3:0:0.4	2.47	40.73	3.28
T-2	1	2	1	3	1:1.25:3.75:0.2:0.6	2.45	36.13	3.16
T-3	1	3	2	2	1:1.5:4.5:0.4:0.8	2.42	33.14	3.08
T-4	1	4	3	4	1:2:6:0.6:1.0	2.37	18.37	2.45
T-5	2	1	4	2	1:2:3:0:0.4	2.62	36.94	3.18
T-6	2	2	3	3	1:2:3:0.2:0.6	2.59	34.55	3.12
T-7	2	3	2	4	1:2.5:3.75:0.4:0.8	2.58	30.13	2.98
T-8	2	4	1	1	1:3:4.5:0.6:1.0	2.55	22.09	2.35
T-9	3	1	2	4	1:2:2:0:0.4	2.69	35.54	3.06
T-10	3	2	1	1	1:2.5:2.5:0.2:0.6	2.67	36.62	3.15
T-11	3	3	3	3	1:2.5:2.5:0.4:0.8	2.59	38.26	2.86
T-12	3	4	4	2	1:3:3:0.6:1.0	2.58	19.92	2.34
T-13	4	1	4	1	1:2.5:1.5:0:0.4	2.69	33.80	2.86
T-14	4	2	3	3	1:2.5:1.5:0.2:0.6	2.67	36.57	3.18
T-15	4	3	1	2	1:2.5:1.5:0.4:0.8	2.63	23.15	2.70
T-16	4	4	2	4	1:3:1.8:0.6:1.0	2.58	18.48	2.18
T-17	5	1	1	4	1:3:1:0:0.4	2.81	25.90	2.78
T-18	5	2	4	3	1:3:1:0.2:0.6	2.77	29.25	3.02
T-19	5	3	3	2	1:3.75:1.25:0.4:0.8	2.76	27.29	2.68
T-20	5	4	2	1	1:3.75:1.25:0.6:1.0	2.63	19.43	2.11

Note: The mixing ratio of the microconcrete in the table is the mass ratio, from left to right: cement: barite sand: quartz sand: lime: water.

**Table 4:** Range analysis results of influencing factors.

Level	Density				Compressive Strength				Elastic Modulus			
	A	B	C	D	A	B	C	D	A	B	C	D
1	2.43	2.66	2.62	2.60	34.09	34.58	26.78	30.53	2.79	3.03	2.83	2.75
2	2.59	2.64	2.58	2.59	30.93	34.62	27.34	28.09	2.65	3.13	2.68	2.80
3	2.63	2.60	2.60	2.62	32.59	28.40	29.01	26.95	2.56	2.86	2.86	3.07
4	2.64	2.54	2.63	2.61	28.00	19.66	26.13	24.68	2.43	2.29	2.94	2.79
5	2.74	-	-	-	25.47	-	-	-	2.35	-	-	-
Range value	0.31	0.12	0.05	0.03	8.62	14.96	4.88	6.27	0.44	0.84	0.26	0.28

**Table 5:** Single-factor analysis of variance results.

Consideration Index	Single Factor Significance	Influencing Factors			
		A	B	C	D
Density	F	17.76	4.5	0.13	0.056
	α = 0.1	Significant	Significant	Non-significant	Non-significant
	α = 0.05	Significant	Significant	Non-significant	Non-significant
Compressive strength	F	8.70	2.53	0.79	0.95
	α = 0.1	Significant	Significant	Non-significant	Non-significant
	α = 0.05	Significant	Non-significant	Non-significant	Non-significant
Elastic Modulus	F	9.32	1.64	0.88	1.04
	α = 0.1	Significant	Non-significant	Non-significant	Non-significant
	α = 0.05	Significant	Non-significant	Non-significant	Non-significant

## Data Availability

The data presented in this study are available on request from the corresponding author.
